# Nitrilotriacetic Acid Improves Plasma Electrolytic Oxidation of Titanium for Biomedical Applications

**DOI:** 10.1021/acsami.3c00170

**Published:** 2023-04-11

**Authors:** Sergiy Kyrylenko, Maciej Sowa, Alicja Kazek-Kęsik, Agnieszka Stolarczyk, Marcin Pisarek, Yevheniia Husak, Viktoriia Korniienko, Volodymyr Deineka, Roman Moskalenko, Izabela Matuła, Joanna Michalska, Agata Jakóbik-Kolon, Oleg Mishchenko, Maksym Pogorielov, Wojciech Simka

**Affiliations:** † Biomedical Research Center, Sumy State University, 31 Sanatorna Street, Sumy 40018, Ukraine; ‡ Faculty of Chemistry, Silesian University of Technology, 6 B. Krzywoustego Street, 44-100 Gliwice, Poland; § Institute of Physical Chemistry PAS, M. Kasprzaka Street 44/52, 01-224 Warsaw, Poland; ∥ Institute of Atomic Physics and Spectroscopy, University of Latvia, 3 Jelgavas Street, Riga LV-1004, Latvia; ⊥ Ukrainian-Swedish Research Center SUMEYA, Sumy State University, 31 Pryvokzalna Street, Sumy 40018, Ukraine; # Faculty of Science and Technology, Institute of Materials Engineering, University of Silesia, 75 Pułku Piechoty Street 1a, 41-500 Chorzów, Poland; ¶ Nano Prime LTD, 25 Metalowców Street, 39-200 Dębica, Poland; ∇ Zaporizhzhia State Medical University, 26 Maiakovskyi Avenue, 69035 Zaporizhzhia, Ukraine

**Keywords:** dental implant, surface processing, plasma electrolytic oxidation, complexing agent, corrosion resistance, biocompatibility

## Abstract

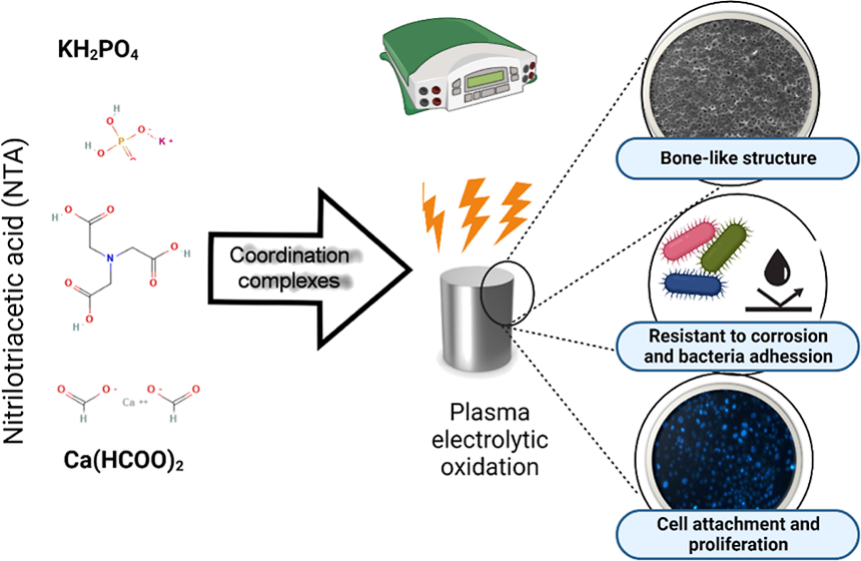

Dental implants have become a routine, affordable, and highly reliable technology to replace tooth loss. In this regard, titanium and its alloys are the metals of choice for the manufacture of dental implants because they are chemically inert and biocompatible. However, for special cohorts of patients, there is still a need for improvements, specifically to increase the ability of implants to integrate into the bone and gum tissues and to prevent bacterial infections that can subsequently lead to peri-implantitis and implant failures. Therefore, titanium implants require sophisticated approaches to improve their postoperative healing and long-term stability. Such treatments range from sandblasting to calcium phosphate coating, fluoride application, ultraviolet irradiation, and anodization to increase the bioactivity of the surface. Plasma electrolytic oxidation (PEO) has gained popularity as a method for modifying metal surfaces and delivering the desired mechanical and chemical properties. The outcome of PEO treatment depends on the electrochemical parameters and composition of the bath electrolyte. In this study, we investigated how complexing agents affect the PEO surfaces and found that nitrilotriacetic acid (NTA) can be used to develop efficient PEO protocols. The PEO surfaces generated with NTA in combination with sources of calcium and phosphorus were shown to increase the corrosion resistance of the titanium substrate. They also support cell proliferation and reduce bacterial colonization and, hence, lead to a reduction in failed implants and repeated surgeries. Moreover, NTA is an ecologically favorable chelating agent. These features are necessary for the biomedical industry to be able to contribute to the sustainability of the public healthcare system. Therefore, NTA is proposed to be used as a component of the PEO bath electrolyte to obtain bioactive surface layers with properties desired for next-generation dental implants.

## 1 Introduction

Successful osseointegration, “a process whereby clinically asymptomatic rigid fixation of alloplastic materials is achieved and maintained in bone during functional loading”,^1^ is a primary target during the development of dental and orthopedic implants. Various techniques have been used to improve the bioactive properties of the implant surfaces and to enhance osseointegration, e.g., blasting with ceramic particles/acid etching, titanium plasma spraying, electrochemical anodization, calcium phosphate coatings, etc.^2^ Despite numerous new strategies for surface modification, only a few of them have been proven to be reproducible, of low cost, and resulted in significant effects in bone ingrowth and osseointegration.

Plasma electrolytic oxidation (PEO) is a method of choice to supply artificial dental/bone implants with bioactive surfaces to improve postoperative healing and long-term stability.^3,4^ Indeed, PEO-generated surfaces have been shown to increase osseointegration^5,6^ and possess antibacterial properties.^7,8^ Moreover, PEO surface layers can increase the corrosion of implants, especially with biodegradable materials such as Mg alloys.^9^ PEO processing could be especially useful for implants in patients with low bone mineral density (osteoporosis) and other medical conditions.^10^ PEO was also used to synthesize a bioactive glass-based coating on titanium (Ti) implants that enhanced their tribological properties with greater corrosion resistance.^11^ PEO was also used to enhance anticorrosive properties of low-carbon steel pipes in the shipping industry.^12^ PEO processing resembles a traditional anodization in which a dielectric layer of oxides is formed. However, the voltage for the PEO is chosen to be high enough to break the dielectric barrier and to establish a dynamic process, in which high-temperature sparks lead to the formation of relatively thick and porous oxide layers with complex surface topography. In particular, such layers can be supplied with various bioactive components, for example, hydroxyapatite (HA), the main mineral component of bone tissues, to enhance osseointegration.^13^

The properties and composition of PEO layers can be modified by adjusting the content of the PEO bath electrolyte and the electrochemical parameters of the PEO process.^14^ The main target in the elaboration of the bath electrolyte is to provide sufficient deposition of Ca and P to the modified surface layers to increase the bioactive properties of the implants. It is well known that inorganic salts of Ca and P are natural factors that could stimulate osteoblast attachment and pre-osteoblast differentiation.^15^ In this regard, the addition of complexing/chelating agents to the PEO bath electrolyte can increase the incorporation of Ca and P ions into the implant surfaces with substantial improvement of their bioactive properties.

We and others have shown that the addition of complexing/chelating agents such as ethylenediaminetetraacetic acid to the bath electrolyte can improve the predictability and results of PEO processing.^16,17^ Nitrilotriacetic acid (NTA) is another well-known chelating agent used in various industrial and scientific applications,^18^ from the production of cleaning detergents to metal affinity chromatography for protein purification in biotechnology.^15,19^ NTA is considered ecologically favorable compared to other chelating agents, as it biodegrades quickly in wastewater and shows low toxicity for the environment.^20^ Previous reports demonstrated that the PEO processing with NTA-containing electrolytes enabled the generation of highly porous and stable ceramic layers with appropriate corrosion profiles.^21^ Our previous research demonstrated that NTA-based PEO ensured the formation of a bioactive surface with high biocompatibility and increased collagen production.^22^ In addition, we recently used NTA to form coordination complexes with ions of metals to enhance the effectiveness of the PEO process on Ti implants.^23^ The preliminary results were published in a separate paper.^23^ In the present report, we present a more detailed investigation of the intrinsic properties of the PEO surfaces with complementary methods, including the roughness assay, Raman spectroscopy, X-ray photoelectron spectroscopy, X-ray diffraction, cross section with energy dispersive X-ray (EDX) mapping, electrochemical corrosion assay, long-term Ringer corrosion assay, cell viability assay, and bacterial adhesion assay. Overall, this comprehensive study demonstrates that NTA as a component of the PEO bath electrolyte provides a substantial effect on the structural and chemical patterns of the surface layers on titanium implants and improves their bioactive properties.

## 2 Results

### 2.1 Surface Physicochemical Properties

**Figure 1:**
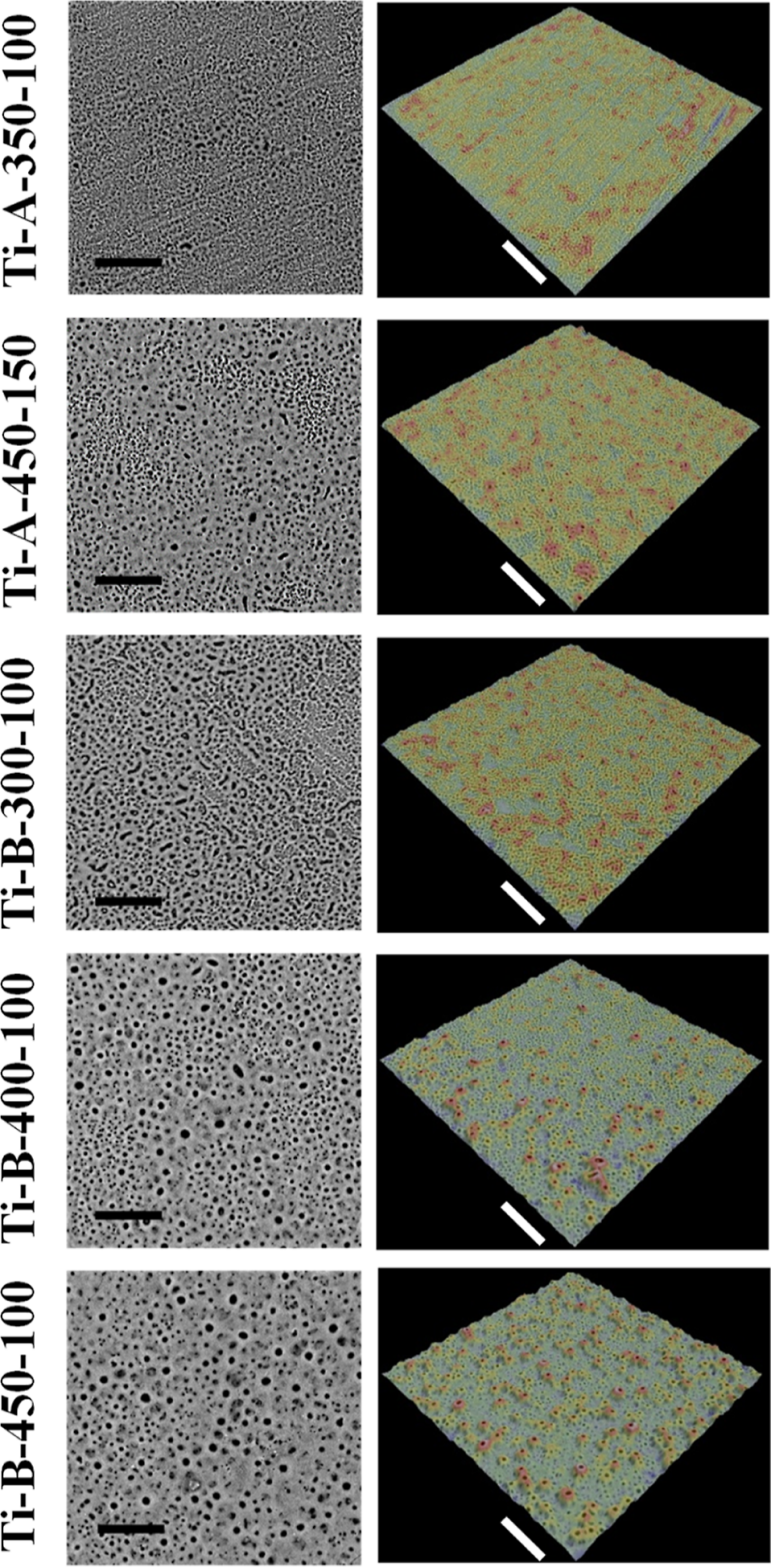
SEM images (left panels) demonstrate increasingly rich surface morphology abundant with pores by the increment in the processing parameters for both bath electrolytes A and B as indicated, while the right panels show 3D analysis of the corresponding PEO surfaces, in which blue color signifies flat or depressed areas, and protrusions are indicated in red. Scale bars = 30 μm.

Preliminary investigations on the morphology and structural properties of the surfaces generated by the PEO conditions described in this article were published in a separate article.^23^ Here, we present a detailed investigation of the intrinsic properties of the same PEO surfaces. Figure 1 shows representative scanning electron microscopy (SEM) images and 3D rendering of the PEO surfaces tested. The average surface roughness parameters Rz and Ra are shown in Table 1. The unevenness parameter Rz denotes the distance between the highest protrusion and the lowest depression on the surface, while the parameter Ra signifies its average roughness.^24^ The data showed that the composition of electrolyte B gave a higher roughness, indicating a more developed surface morphology. Details of the profiling of the sample Ti-B-450-100 with the highest unevenness profile are shown in Supporting Information, Figure S1. In conclusion, the increase in voltage resulted in a higher roughness of the PEO surface layers. The Ti-B-450-100 sample had the highest unevenness. There was a gradual increase in the unevenness with the increment in the processing parameters for both bath electrolytes A and B. This suggests that the PEO with both electrolytes is able to generate surfaces with a wide range of controllable properties. It was shown that the moderately rough surfaces (Sa > 1–2 μm) displayed stronger bone responses than the surfaces with a rougher morphology.^25^ Accordingly, the sample Ti-B-400-100 appears to be able to produce a surface with the unevenness optimal for osseointegration. However, the structure and composition and the PEO surfaces still require further studies to relate their properties to osseointegration and long-term stability of the implants, as well as their usability in other biomedical applications.

**Table 1:** Roughness (Parameters of Unevenness Rz and Ra) and Thickness of PEO Oxide Layers of Selected PEO Samples

sample	Ti-A-350-100	Ti-A-450-150	Ti-B-300-100	Ti-B-400-100	Ti-B-450-100
Rz, μm	3.39 ± 0.67	4.59 ± 0.10	4.31 ± 0.36	6.97 ± 1.90	8.91 ± 0.73
Ra, μm	0.68 ± 0.11	1.1 ± 0.08	1.00 ± 0.09	1.72 ± 0.47	2.14 ± 0.10

**Figure 2:**
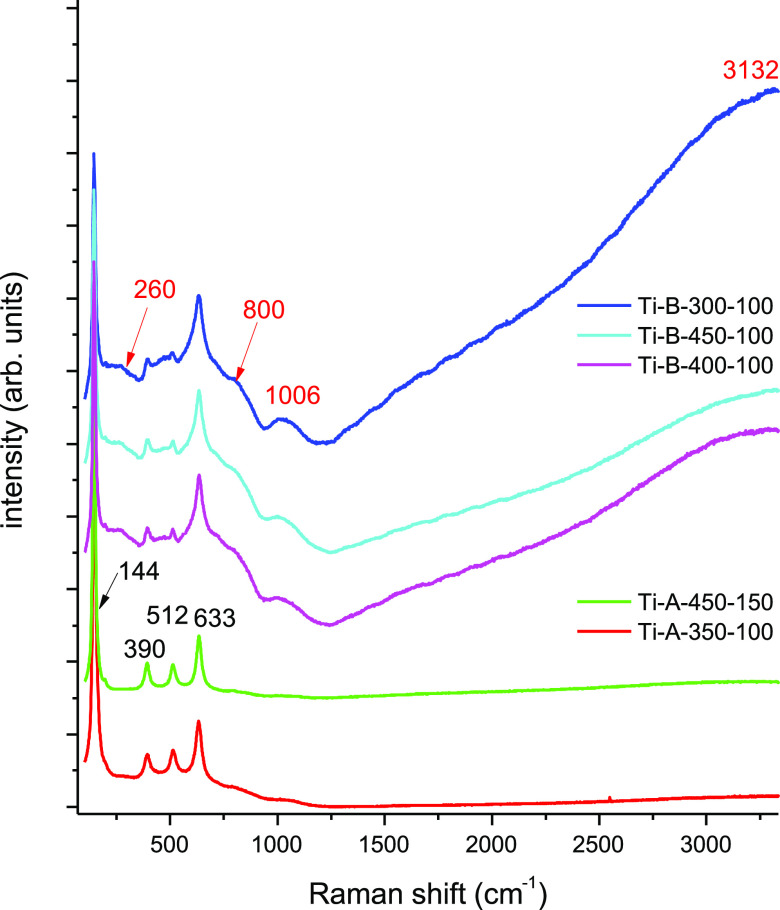
Raman spectra showing the presence of anatase (narrow signals at 144, 390, 512, and 633 cm^–1^) and phosphate groups (broad signals at 260, 800, 1006, and 3132 cm^–1^) of the tested samples.

Raman spectra showed that well-crystallized TiO_2_ was present on the surfaces of all samples tested (Figure 2). Well-crystallized TiO_2_ was present in the form of anatase. Furthermore, several samples displayed broad signals from phosphate groups, indicating the presence of phosphates in the amorphous phase (calcium phosphate). Various samples showed different phosphate content. The content of phosphate groups in relation to titanium dioxide was estimated by determining the ratio *I*_1006_/*I*_144_ (Table 2). The lowest phosphate content was recorded for the Ti-B-400-100 sample and the highest for Ti-B-450-100. No additional signals from NTA or products of its transformation were recorded during the PEO processing. Overall, the Raman spectra illustrate the changes in calcium phosphate content in individual samples, depending on the electrolyte and the electrochemical parameters used, and suggest that PEO treatment can lead to the generation of osteogenic surfaces due to the introduction of the controllable content of the surface phosphate groups.

**Table 2:** Comparison of the Phosphate Content on the Basis of the Intensity of Phosphate Vibration 1006 cm^–1^ to TiO_2_ 144 cm^–1^ with No Additional Signals from NTA Recorded

sample	Ti-A-350-100	Ti-A-450-150	Ti-B-300-100	Ti-B-400-100	Ti-B-450-100
*I*_1006_/*I*_144_	0.01190 ± 0.00016	0.00327 ± 0.00008	0.04222 ± 0.00014	0.00130 ± 0.0007	0.05212 ± 0.00015

**Figure 3:**
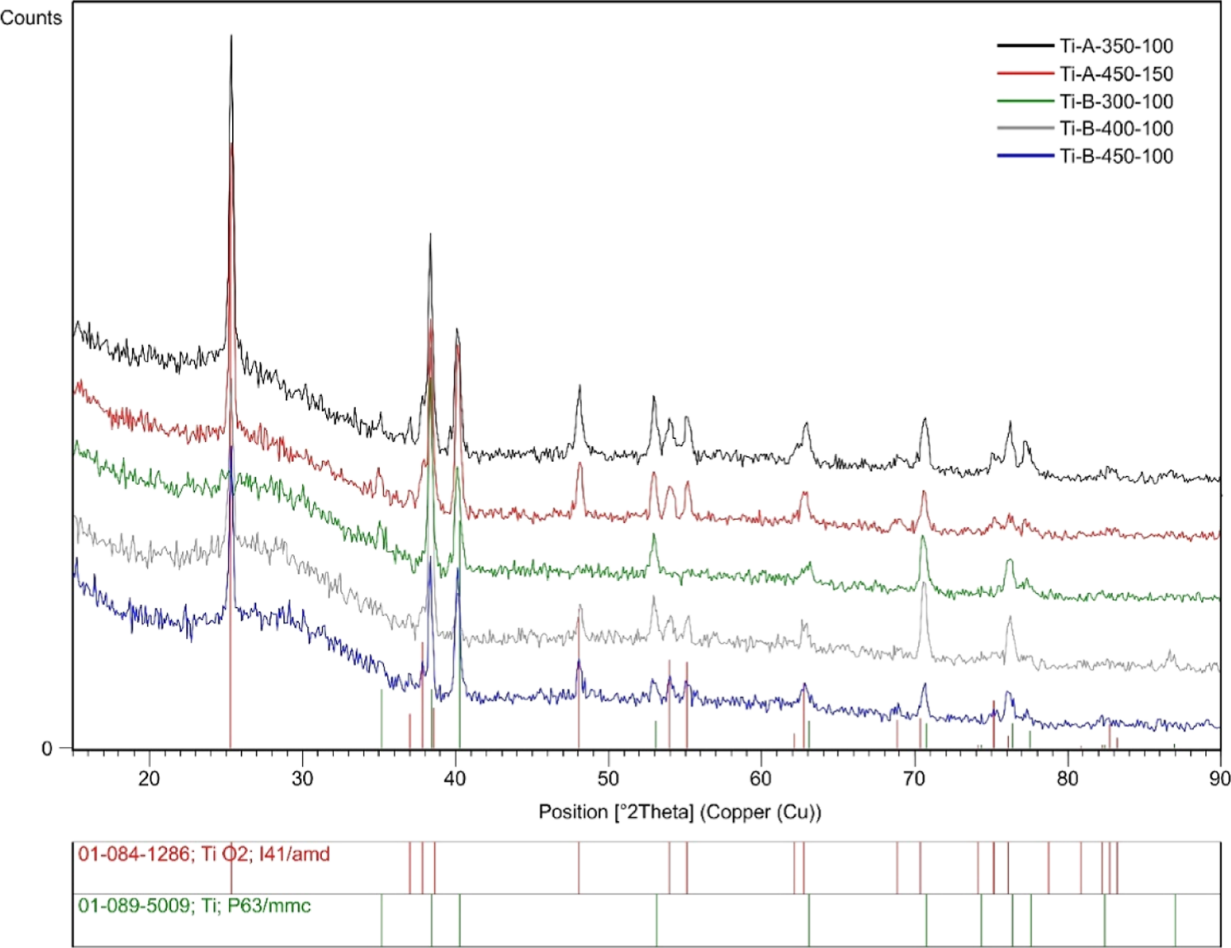
TL-XRD spectra of the surface of the anodized samples.

Thin-layer diffraction (TL-XRD) analysis of the PEO oxide layers showed that they all had an amorphous-crystalline nature, regardless of the parameters of the anodic oxidation process (Figure 3). Each spectrum in the angular range of 20–30° shows a “halo” characteristic of amorphous materials with various heights, as shown for the Ti-B-400-100 sample. In the crystalline phase, mainly titanium oxide in the form of anatase was detected in the samples Ti-A-350-100, Ti-A-450-150, Ti-B-300-100, and Ti-B-450-100 (Figure 3). Little or no rutile was detected in all PEO layers studied.

**Figure 4:**
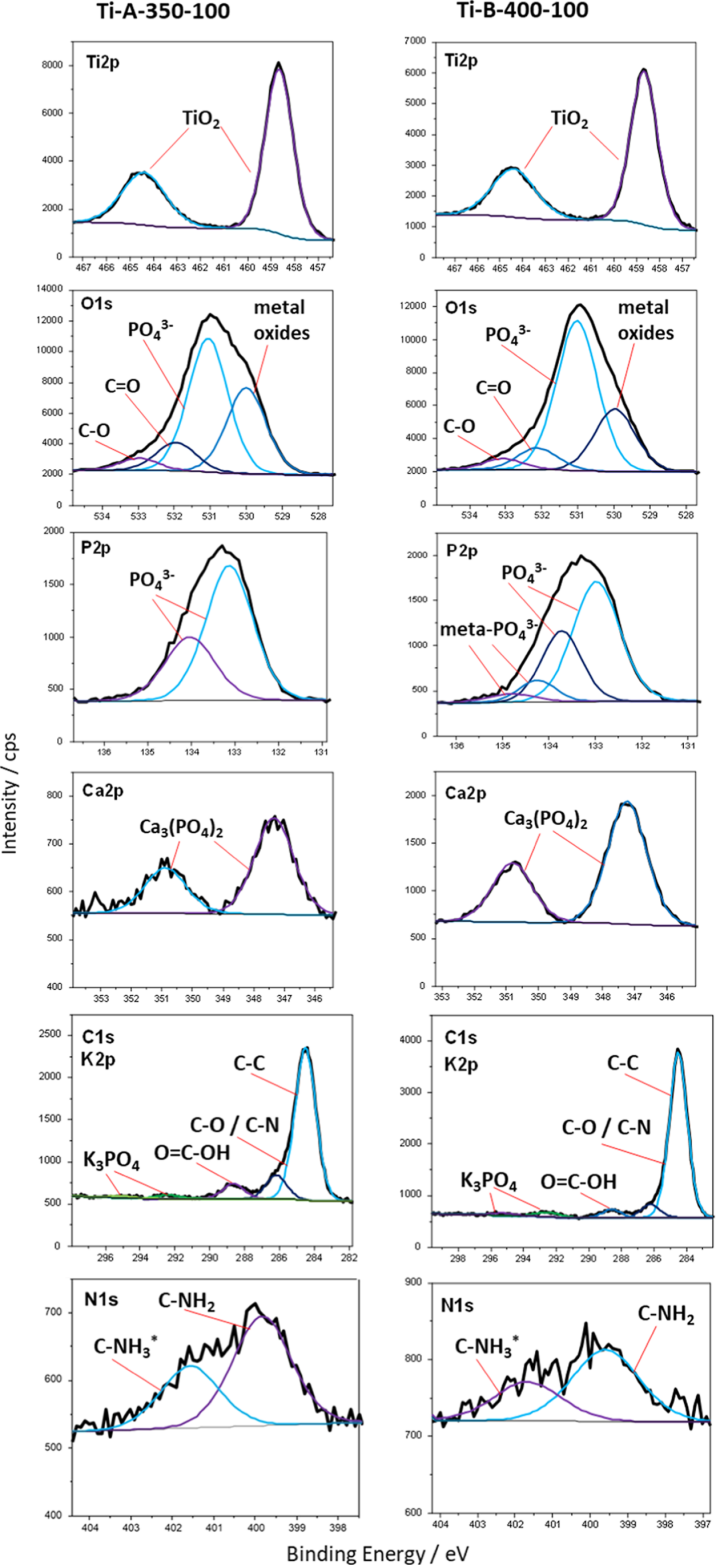
XPS spectra of Ti 2p, O 1s, P 2p, Ca 2p, C 1s, K 2p, and N 1s. The PEO oxide layer is composed of TiO_2_ that is enriched in Ca_3_(PO_4_)_2_ and functional −NH_2_ groups.

The X-ray photoelectron spectroscopy (XPS) method allows for precise measurement of concentrations of the elements on the tested surfaces and assigning of the chemical states to the elements. In all samples tested, only elements related to the process were detected (Table 3; complete set of data is in Supporting Information, Tables S1 and S2) with no nonrelated contaminations except minor amounts of Si, which remained after preprocessing of the samples (grinding). The highest concentration of oxygen (46 to 53 at. %) was recorded on the surfaces of the samples. Oxygen was present mainly in the form of titanium oxide but also in the form of phosphate and carbon compounds, as shown in Figure 4 (O 1s spectra). This is suggested by two factors: the shape of the O 1s peak and the matching of individual maxima at binding energies (BE) of about 530.0, 531.0, 532.0, and 533.0 eV. In addition, the positions of the main spectral line maxima for Ti 2p, P 2p, and C 1s confirm this, where their positions can be assigned to TiO_2_ (458.7 eV), PO_4_^3–^ (133.0 eV), and carbon and oxygen functional groups (286.2 and 288.6 eV) because compounds of oxygen and carbon originated from NTA, the complexing agents used during oxidation. Therefore, the phosphorus concentration was within the range of 10–11% and present only in the form of phosphate: calcium or potassium (Tables S1 and S2). This element comes entirely from the components of the bath electrolyte. Another important element, Ca, was present in each of the samples tested. Its concentration ranged from 0.5 to 2.8 at. %. Calcium was present only in the form of calcium phosphate, formed during the anodic oxidation of titanium. Its source was Ca–NTA complexes. Potassium was present in small amounts, up to 0.2 at. % in the form of phosphate from the bath component. Titanium was detected in the oxide form at a concentration of about 10 to 15 at. %. Interestingly, the presence of nitrogen was also detected. Its source was the Ca–NTA complexes of the bath electrolyte. The nitrogen concentration was relatively low, not exceeding approximately 1.5 at. %, in the form of amine groups linked to carbon (Figure 4). This combination of chemical composition of the oxide layer may favor the intensification of osseointegration processes. The obtained test results agree with the test results obtained with the EDX, XRD, and Raman methods.

**Table 3:** Chemical Composition—Atomic Fractions (%) of Elements in the Selected PEO Coatings Determined by XPS

	elements, at. %						
sample	P	C	K	Ca	N	Ti	O
Ti-A-350-100	10.0	19.2	0.1	0.5	1.6	14.6	54.1
Ti-B-400-100	10.7	27.1	0.3	2.8	1.1	10.4	47.6

**Figure 5:**
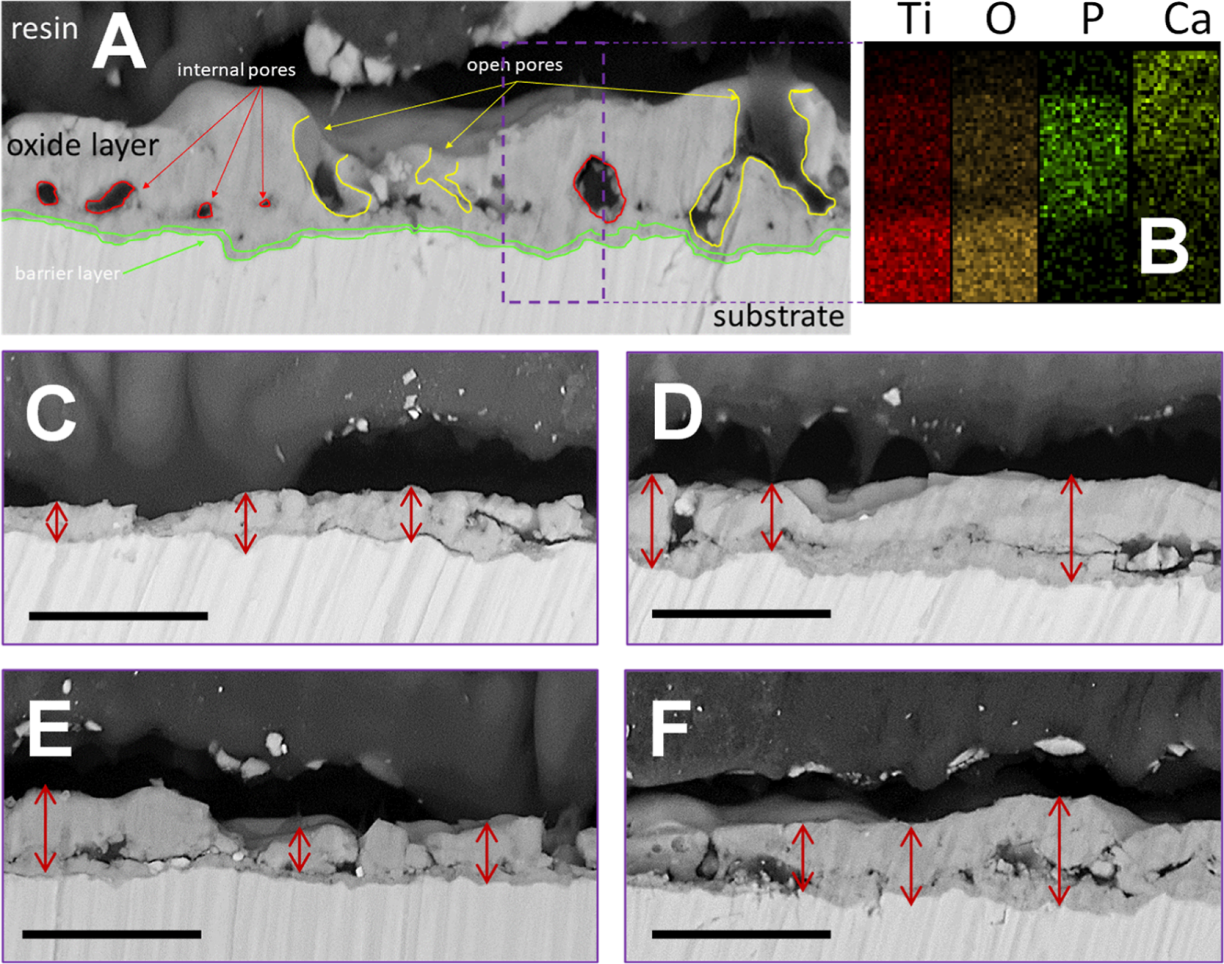
Cross-sectional analysis of the PEO coatings. (A,C–F) SEM images of the cross section, in which Ti is seen as a light area in the bottom part and epoxide resin as a dark upper part with the PEO layer between them. Red arrows indicate the locations of the measurements of the thickness of the PEO layers. (A) sample Ti-B-450-100; (C) sample Ti-A-350-100; (D) sample Ti-A-450-150; (E) sample Ti-B-300-100; (F) sample Ti-B-400-100; scale bars = 10 μm. (B) EDX mapping for the Ti-B-450-100 sample, where distribution of titanium, oxygen, phosphorus, and calcium in the oxide layer is visible.

The obtained structure of the oxide layers on titanium is typical for PEO coatings (Figure 5). The layer consists of three sublayers. The first, in direct contact with the titanium, is a dense barrier oxide layer (Figure 5A). The second is a layer containing closed pores (Figure 5A). The third outermost one contains open pores (Figure 5A). Moreover, cross-sectional analysis revealed that the PEO layers had pores, which appeared interconnected.

Analysis of the cross sections of the PEO layers produced on Ti and their EDX mapping revealed that the PEO coatings adhered well to the titanium regardless of the processing parameters used (Figure 5). Any visible layer detachment was an artifact of the sample preparation procedure. The thicknesses of the oxide layers for the Ti-A samples were 1.92 ± 0.75 and 3.96 ± 0.95 μm for the Ti-A-350-100 and Ti-A-450-150 and for the Ti-B samples, were 3.28 ± 0.81, 6.68 ± 1.84, and 6.2 ± 1.34 μm for Ti-B-300-100, Ti-B-400-100, and Ti-B-450-100, respectively. Therefore, the thickness of the coatings did not exceed 7 μm. Notably, the formation of the PEO oxide layer depended on the voltage applied, where increasing the voltage generally led to increased thickness (this report and ref (17)). The EDX technique was used to determine the content of the selected elements (Figure 5B for the sample Ti-B-450V-100; the EDX data for other samples are shown in Supporting Information, Figure S2). The atomic ratios of Ca/P, Ti/P, and Ti/Ca of the oxide layers are shown in Table 4.

**Table 4:** Atomic Ratios in Oxide Layers

	ratios of at. % concentrations		
sample	Ca/P	Ti/P	Ti/Ca
Ti-A-350V-100	0.34	6.42	18.66
Ti-A-450V-150	0.13	6.98	52.33
Ti-B-300V-100	0.22	5.26	23.50
Ti-B-400V-100	0.32	6.54	20.14
Ti-B-450V-100	0.38	8.17	21.29

Data confirmed that in each sample the components of the electrolyte bath (Ca and P) were incorporated into the entire volume of the oxide layers (Figure 5B). The content of Ti, Ca, and P in the oxide layers did not show any consistent dependence on the voltage. Neither of the Ca/P ratios showed any consistent dependence on the voltage. With an increase in the current, a decrease in the Ca/P ratio was noted for electrolyte A, while an increase was observed for electrolyte B. These results suggest that PEO can be used to generate oxide coatings enriched in both Ca and P, the constituents of hydroxyapatite, which is the main mineral component of bone tissue; therefore, PEO processing can be used to form surfaces with increased osseointegrative capacity.

### 2.2 Corrosion Investigations

#### 2.2.1 Electrochemical Corrosion Assay

The corrosion resistance of titanium before and after electrochemical treatment was assessed by both direct current (open-circuit potential—OCP; linear polarization resistance—LPR; potentiodynamic polarization—PDP) and alternating current (electrochemical impedance spectroscopy—EIS) methods (Tables 5 and 6, respectively). Potentials are reported with respect to saturated calomel electrode (SCE). The PDP curves and impedance spectra corresponding to the analyzed samples can be reviewed in Figures S3 and S4, respectively.

**Table 5:** Summary of the Results Obtained by Means of Direct Current Methods for Testing the Corrosion Resistance of Titanium before and after Electrochemical Treatment

sample	*E*_OCP_, mV vs SCE	*E*_cor,LPR_, mV vs SCE	*E*_cor,PDP_, mV vs SCE	*R*_p_, MΩ•cm^2^	*i*_pas_, nA•cm–^2^
polished Ti	–39.7 ± 39.5	–30.3 ± 34.2	–77.4 ± 35.8	6.91 ± 0.34	407 ± 22
Ti-A-350-100	303 ± 11	316 ± 12	179 ± 17	39.8 ± 1.9	3.96 ± 0.51
Ti-A-450-150	336 ± 25	330 ± 23	200 ± 23	7.99 ± 0.16	8.44 ± 0.42
Ti-B-300-100	238 ± 23	231 ± 12	125 ± 16	48.4 ± 0.30	2.42 ± 0.68
Ti-B-400-100	291 ± 25	282 ± 13	153 ± 23	24.9 ± 1.6	4.04 ± 0.73
Ti-B-450-100	301 ± 4	295 ± 10	187 ± 14	14.4 ± 1.9	6.61 ± 0.39

**Table 6:** Summary of the Results Obtained by Means of EIS for Testing the Corrosion Resistance of Titanium before and after Electrochemical Treatment

sample	polished Ti	Ti-A-350-100	Ti-A-450-150	Ti-B-300-100	Ti-B-400-100	Ti-B-450-100
*R*_0_, Ω·cm^2^	26.6 ± 0.3	20.9 ± 3.6	27.4 ± 1.4	32.4 ± 0.3	25.7 ± 1.1	28.7 ± 1.3
*Q*_1_, s^*n*^·MΩ^–1^·cm^–2^		0.50 ± 0.06	0.21 ± 0.04	0.11 ± 0.01	0.10 ± 0.00	0.31 ± 0.03
*n*_1_		0.82 ± 0.00	0.89 ± 0.01	0.94 ± 0.01	0.93 ± 0.02	0.86 ± 0.01
*R*_1_, kΩ·cm^2^		46.2 ± 3.0	49.7 ± 36.4	468 ± 18	153 ± 7	8.42 ± 2.76
*Q*_2_, s^*n*^·MΩ^–1^·cm^–2^		4.49 ± 0.34	4.75 ± 1.87	1.63 ± 0.22	2.04 ± 0.46	8.11 ± 0.55
*n*_2_		0.82 ± 0.00	0.75 ± 0.02	0.72 ± 0.01	0.65 ± 0.09	0.65 ± 0.00
*R*_2,_ kΩ·cm^2^		89.6 ± 4.3	44.0 ± 24.2	371 ± 5	219 ± 84	14.3 ± 7.7
*Q*_3_, s^*n*^·MΩ^–1^·cm^–2^	24.0 ± 4.9	4.20 ± 0.63	6.26 ± 0.69	7.91 ± 1.05	7.60 ± 1.44	5.28 ± 0.90
*n*_3_	0.93 ± 0.01	0.81 ± 0.00	0.81 ± 0.00	0.91 ± 0.02	0.83 ± 0.02	0.87 ± 0.05
*R*_3_, MΩ·cm^2^	1.73 ± 0.96					
χ^2^	<8.46 × 10^–4^	<3.10 × 10^–4^	<3.20 × 10^–4^	<1.28 × 10^–3^	<9.24 × 10^–4^	<4.73 × 10^–4^

The data showed that the values of the OCP (stationary, free) observed after an hour of immersion differed from *E*_cor,LPR_ and *E*_cor,PDP_. This effect is especially strong for the PDP curve. This is due to the charging current of samples with a significant current capacity caused by the potential sweep operation. This current does not correspond to Faradaic reactions and is therefore an undesirable phenomenon because it skews the values of the measured potentials. The effect of registering the additional share of the charging current is a change in the corrosion potential *E* (*i* = 0) toward more negative values. In this study, we define *i*_pas_ as the passivation current density, and it was the value of the current density measured at the terminal point of the PDP curve. The lower its value, the better is the ability of the coating to limit the flow of current. In other words, the coating is more resistant to corrosion in oxidative environments. Based on the results presented in Tables 5 and 6, it can be concluded that, in general, the best corrosion resistance was obtained at a lower voltage of electrochemical oxidation (e.g., 300 V). The applied current density during oxidation had a less pronounced influence on the protective properties of the oxide layers.

The results obtained using direct current methods (OCP, LPR, and PDP) are generally consistent with those obtained with EIS (Table 6). The EIS parameters have been obtained by fitting the obtained spectra (Figure S4) to the equivalent electrical circuits (Figure S5). In the case of the polished Ti sample, a simple *R*_0_(*Q*_3_*R*_3_) circuit has been used. *R*_0_ is the solution resistance between the reference electrode (RE) tip and the working electrode (WE) surface. *Q*_3_ represents the non-ideal double-layer capacitance in the form of a constant-phase element. *R*_3_ is the charge-transfer resistance of the corrosion reaction. For the electrochemically treated samples, a more complicated *R*_0_(*Q*_1_(*R*_1_(*Q*_2_(*R*_2_(*Q*_3_*R*_3_))))) circuit was utilized. The additional two parallel *RQ* pairs represented the resistance and non-ideal capacitance of the outer porous (1) and inner barrier (2) oxide layers (Figure S5). From the shape of the corresponding spectra (Figure S4), it can be deduced that the low-frequency part of the plots is dominated by the capacitive behavior which makes it impossible to properly assess the charge-transfer resistance. For the sake of obtaining the remaining circuit parameters values, it was assumed that *R*_3_ is approaching infinity (*R*_p_ in Table 5 for the oxidized specimens was very high, which supports this notion). It is the reason for the lack of data corresponding to *R*_3_ for the electrochemically treated Ti samples in Table 6.

For samples with high polarization resistance and low passivation current density (Ti-A-350-100 and Ti-B-300-100), the lowest values of *Q*_3_ and values of *n*_3_ closest to unity were observed. The favorable values of the parameters are also accompanied by relatively high values of the resistances *R*_1_ and *R*_2_, which suggest good tightness of the layers and a small number of defects inside which the corrosion current could occur. These parameters were observed to decrease with the increasing oxidation voltage, as can be seen for bath B. As the value of this process parameter increased, the value of *Q*_3_ decreased from 7.91 s^*n*^ MΩ^–1^ cm^–2^ for the sample oxidized at 300 V to 7.60 s^*n*^ MΩ^–1^ cm^–2^ for sample Ti-B-400-100 up to 5.28 7.91 s^*n*^ MΩ^–1^ cm^–2^ when the oxidation was carried out at 450 V. We concluded that all samples tested showed a higher corrosion resistance than that for the titanium reference sample. The highest corrosion resistance was obtained for the Ti-B-300-100 modification (the highest value of the *n*_3_ parameter with a simultaneously low *Q*_3_).

#### 2.2.2 Long-Term Ringer Corrosion Assay

**Figure 6:**
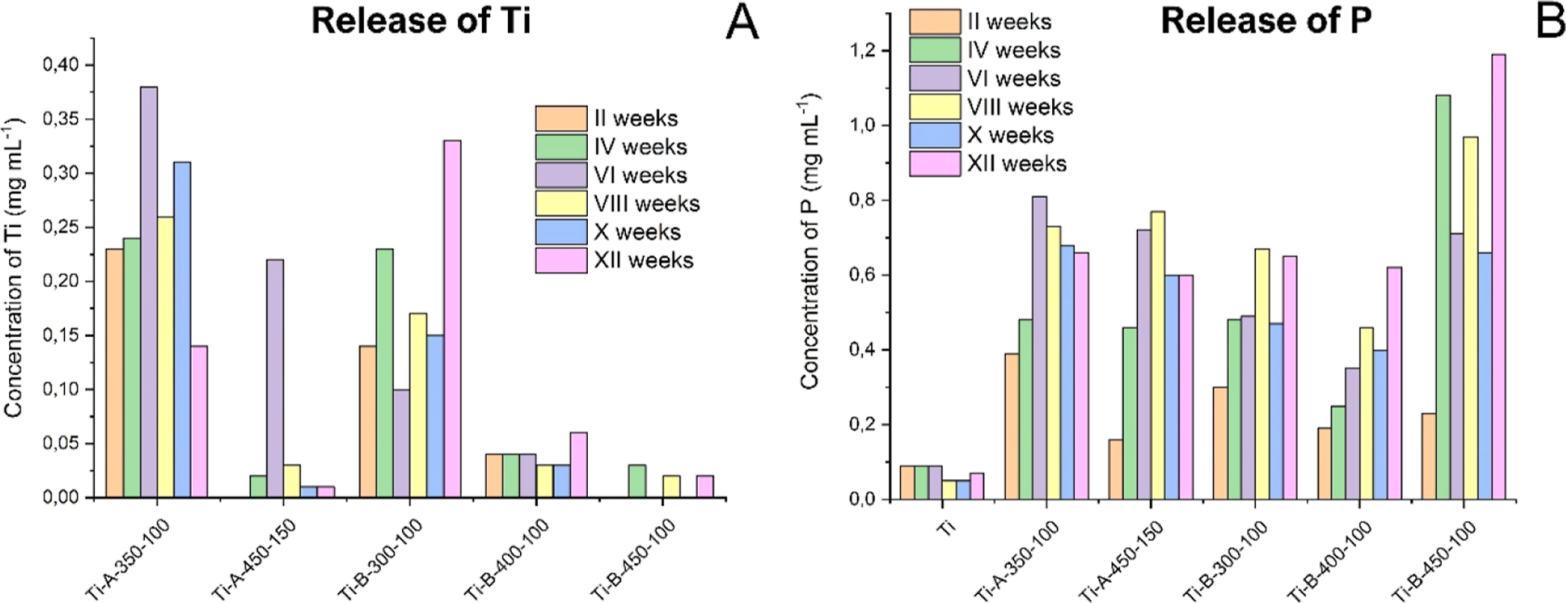
Release of selected elements from the PEO surfaces formed on the titanium specimens during prolonged 12 weeks incubation in Ringer solution. (A) Release of Ti; (B) release of P, where minuscule amounts of the P in the Ti sample were due to the unspecific background of the assay (release of Ca is not shown since background concentration of Ca in Ringer solution did not allow to detect consistent elevation of Ca ions released from the specimens). Ti signifies the non-PEO treated, polished titanium sample, and for this sample, concentration of Ti ions released to Ringer solution was below 0.01 mg mL^–1^. The difference in the values between is shown for the qualitative evaluations only, and no significant difference was found.

In line with our previous observation,^14^ the control Ti sample did not release Ti ions into the corrosion medium even after 3 months of incubation at 37 °C (Figure 6). However, the PEO-treated samples release Ti already after the initial period of the experiment (2 weeks). Titanium is known to be a highly inert material. However, its surface is hydrophobic and requires effort to increase its biocompatibility. PEO can dramatically increase biocompatibility, leading to increased osteoconductivity. However, during PEO processing, since Ti becomes oxidized, it is transformed into compounds which can slowly dissolve in body fluids. The sample Ti-B-300-100 showed the highest amount of released Ti, while the sample Ti-B-450-100 was the least prone to release Ti. Therefore, the PEO surface of the sample Ti-B-450-100 can be considered as an optimal surface layer for biomedical applications in terms of Ti release. Interestingly, the release of P from the PEO layers showed a trend to increase with higher electrochemical parameters and longer incubation in Ringer solution. In particular, phosphates can promote the formation of hydroxyapatites, which further proves that PEO processing can generate surface layers with osteoconductive capacity. The dynamics of the Ca release was also investigated. However, since Ringer solution contains Ca at relatively high concentrations (higher than the presumed released Ca ions), the assay did not detect a consistent elevation of Ca in Ringer’s background (not shown). However, the Ti-B-300-100 sample showed the trend to release the least amount of Ca, while the sample Ti-B-450-100 was relatively high with the release of Ca. Moreover, this sample was the most active in releasing P. In conclusion, an increase in biocompatibility comes with a price, and increasing both biocompatibility and corrosion resistance requires a delicate balance and fine tuning of the protocols to obtain bioactive surfaces.

### 2.3 In Vitro Characterization

#### 2.3.1 Cell Viability Assay

**Figure 7:**
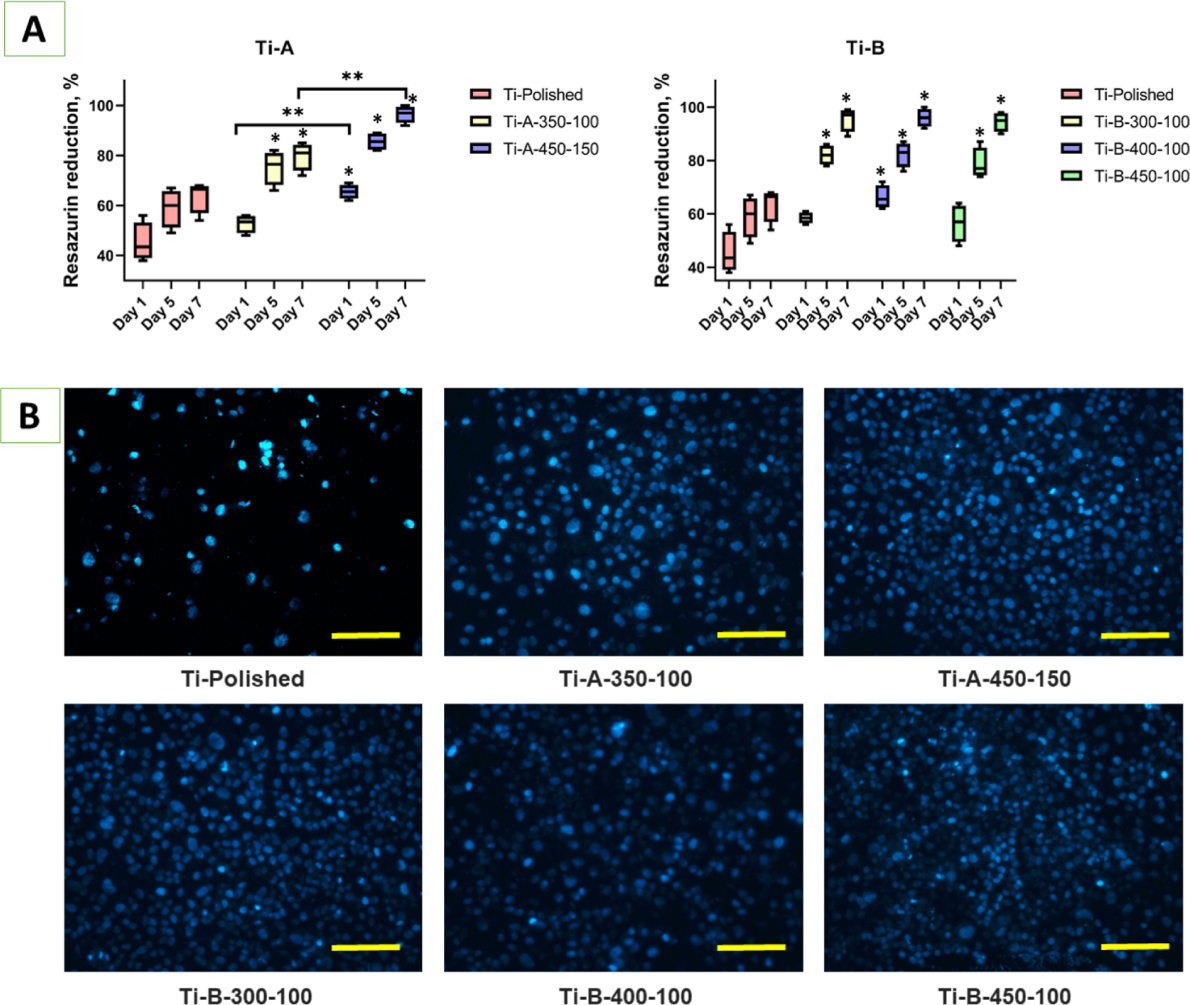
U2OS rat osteoblasts grown on Ti samples with PEO surfaces. (A) Graphical representation of resazurin reduction assay for cell proliferation on Ti samples from day 1 to 7, where the value of resazurin reduction in wells with the cells only was taken as 100%. (B) Fluorescent DAPI staining on day 7. *significant (*p* ≤ 0.05) difference between PEO-treated and control groups; **significant (*p* ≤ 0.05) difference between PEO-treated groups; scale bar = 200 μm.

Efficient cell attachment was observed within 24 h in all experimental groups (Figure 7). However, Ti-A-450-150 and Ti-B-300-100 demonstrated significantly better osteoblast attachment and proliferation on day 1 compared to the polished Ti sample (Figure 7A). Osteoblast cultivation for 7 days showed appropriate cell proliferation with viability above 70% that meets the ISO 10993: 5 criteria for biocompatible surfaces. Moreover, the cells showed significantly better proliferation on the Ti samples with PEO surfaces obtained in both bath electrolytes, which reach 100% for most surfaces. Samples Ti-A-350-100 and Ti-A-450-150 did not demonstrate any significant difference between groups, while Ti-A-450-150 showed slightly better cell growth compared to Ti-A-350-100. Our previous data supported the hypothesis about the simultaneous effect of surface roughness and chemical patterns for cell attachment and proliferation.^26,27^ Despite the similar chemical structure, the Ti-A-450-150 sample had more developed porous surface morphology with higher distances between the highest protrusion and the lowest depression on the surface (unevenness parameter Rz) and higher average roughness (parameter Ra), which could provide a better environment for cell proliferation.

4′,6-diamidine-2′-phenylindole dihydrochloride (DAPI) staining (Figure 7B) demonstrates substantially higher cell confluence on all PEO-treated surfaces compared to polished Ti. The PEO-treated surfaces were completely covered with cell nuclei except for the Ti-A-350-100 sample, which demonstrated some nuclei-free areas. In conclusion, the cell culture assay demonstrated that all Ti samples showed high biocompatibility with significantly higher cell proliferation in the PEO-treated groups.

#### 2.3.2 Bacterial Adhesion Assay

**Figure 8:**
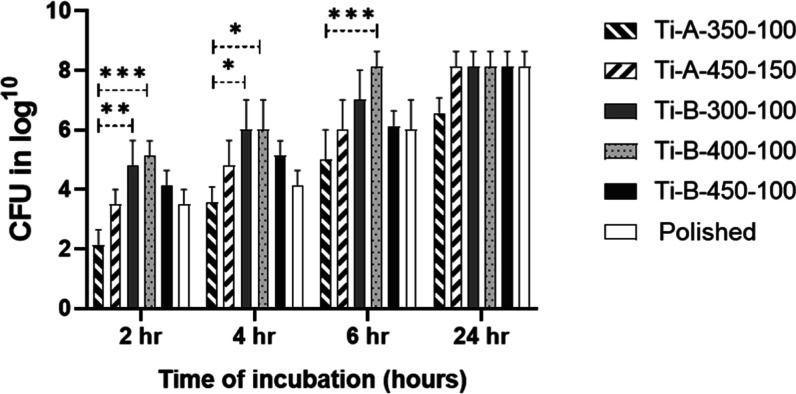
Number of bacterial cells (in log 10 CFU) adhered to Ti with various PEO coatings and to polished Ti surfaces at different time-points of incubation in the bacterial suspension; *p < 0.05; **p < 0.01; ***p < 0.001.

Both polished Ti and the PEO-generated surfaces showed the ability to adhere bacteria already at the initial time points of incubation in the bacterial suspension (Figure 8). The Ti-A-350-100 surface of PEO showed minimum adhesion of bacteria. With time, the adhesion to all surfaces increased gradually, reaching the maximum at 24 h. However, the bacterial antiadhesion properties of the sample Ti-A-350-100 were consistently higher even in comparison to the reference sample, including at the 24 h time point. This suggests that the development of the most optimal protocol for manufacturing dental implants with high osteogenic properties while also being resistant to bacterial colonization can rely on the PEO methods using the NTA-containing bath electrolyte. However, further studies to find a delicate balance between the ability to support high proliferation rate for eukaryotic cells and low adherence to bacteria are needed.

## 3 Discussion

Integration of bone and gum tissues with the implant depends on the characteristics of its surface (reviewed, e.g. ref (28)). Furthermore, the stability of dental implants also depends on the health status of the patients.^29^ In addition, often definite conclusions on the success of various implants can not be drawn because of the lack of long-term data, heterogeneity, and variability in study designs and lack of reporting on confounding factors as in the case of, e.g., periodontally compromised patients.^30^ This further emphasizes the importance of the development of new technologies to manufacture dental implants with bioactive surface layers. The term “bioactive” could be applied to a dental implant if it could modulate a natural biological process,^31^ in particular, to be able to support the adhesion and proliferation of the cells. The term was also defined as “in addition to their primary function of restoring or replacing missing tooth structure, they actively stimulate or direct specific cellular or tissue responses, or both, or they can control interactions with microbiological species”.^32^ In this report, we demonstrate that the PEO with the NTA-based bath electrolyte can generate surfaces that might favor the mechanical matching with the bone interface while being capable of stimulating cell adhesion and proliferation, as well as preventing bacteria colonization at the early stage of implantation.

The titanium surface undergoes rapid natural formation of thin oxide films upon contact with atmospheric oxygen. The thicker oxide layers can be deposited artificially by various methods. Anodization is a widely known low-cost and fast electrochemical surface-treatment method used to obtain surfaces with modified properties.^33^ However, bioactive layers on the surfaces of Ti require more sophisticated approaches, and PEO has become a method of choice to increase, e.g., osseointegrative properties of Ti implants. Here, we investigated the effect of NTA as a complex-forming agent in the PEO bath electrolyte. The SEM of the cross sections revealed that the PEO layers are relatively thick and are firmly attached to the underlying metal. Moreover, they showed interconnected pores, the number of which increased with voltage. The increased voltage also resulted in thicker PEO layers. The elements of the bath electrolyte were incorporated into the oxide layers and distributed relatively evenly. Especially important is the incorporation of both Ca and P, the components of hydroxyapatite. Notably, the Ca/P ratio was modified by adjusting parameters of the PEO processing. This can be used as an important method to achieve the desired osseointegrative capacity of surfaces due to selective stimulation of mesenchymal stem cells (MSCs) and pre-osteoblast differentiation. Several studies analyzed the bioactive potential of apatite-formation ability with controversial conclusions,^34^ while Mueller et al. demonstrated that Ca-P surfaces were able to drive differentiation of the MSC to osteoblasts in the absence of osteogenic differentiation supplements in the medium.^35^ Other reports demonstrated that Ca–P substrate triggered osteogenic differentiation through the genes of SMAD and RAS families that were typically regulated during dexamethasone-induced differentiation.^36^ It was in line with other studies, where the formation of abundant nano- and micro-pores was observed for PEO in electrolytes with Ca and P ions^37^ and other additives, particularly Mn and Si.^38^ Moreover, the process of formation of a bioactive coating consisting of titanium oxides and hydroxyapatites in Ti alloys by PEO was recently shown to be effective in molten salts as bath electrolytes.^39^ Interestingly, PEO processing was also applied to control the biodegradation rate and biocompatibility of magnesium implants in experiments in rabbits in vivo.^40^ Our report demonstrated the capacity of the chelating agent to initiate deposition of Ca–P complexes as substrates for the PEO processing, which resulted in surface layers with advanced bioactive parameters. This was in line with previous studies which suggested the ability of inorganic Ca and P to provide the bioactive properties for dental implant surfaces.

Various calcium phosphate content and its dependence on the PEO parameters were confirmed by Raman spectroscopy. The Raman spectra also showed the presence of titanium dioxide in a well-crystalized form. Titanium oxide was the predominant form of oxygen present on the surfaces of the samples, which was revealed by XPS analysis. Oxygen was also present in the form of phosphates and compounds with carbon. The presence of NTA in the bath electrolyte may have contributed to the accumulation of Ca and P in the PEO layers. XPS also detected the presence of nitrogen, and the only possible source for nitrogen was the NTA. Addition of N to the surface layers promoted adsorption of fibronectin and the adhesion, proliferation, and mineralization of human bone marrow MSCs, thus enhancing initial osseointegration.^41^ Thus, nitrogen can be an important component of bioactive surfaces, and modulation of the nitrogen content by addition of NTA and other components can be used in the manufacturing of bioactive surfaces on an industrial scale. TL-XRD analysis detected the amorphous-crystalline nature of the obtained PEO surfaces.

An important feature of the anodization process provides a viable method for modifying the corrosion-resistant and mechanical properties of metals. Ti is known to be exceptionally inert. However, its bare surface is hydrophobic and hence requires modifications to increase its hydrophilicity in order to achieve higher bioactivity. Previously, we showed that the surfaces of Ti become more hydrophilic after PEO in bath electrolytes with complex forming agents.^17,23^ Moreover, PEO dramatically increased the roughness of the surfaces. Roughness is a parameter of a well-developed morphology, and hence an increase in roughness generally leads to better osseointegration.^42^ However, despite decades of progress, the ideal surface roughness for osseointegration still remains unclear.^43^ Moreover, surfaces rich in morphological features may contain compounds that are more prone to dissolve in bodily fluids. The electrochemical corrosion assay in combination with the long-term Ringer corrosion assay showed that a lower PEO voltage is preferred to obtain surfaces with higher corrosion resistance.

The biological properties of the PEO-generated surfaces were investigated in osteoblast assays in cell cultures and in a bacteriological assay. Cell viability and proliferation assays showed that PEO processing dramatically increases the ability of eukaryotic cells to adhere to the surfaces and proliferate on them. This was especially evident after DAPI staining and observation under a fluorescent microscope. When the studies of bioactivity of the calcium- and phosphorus-enriched oxide layers produced are summarized, it can be concluded that most of the PEO layers are highly bioactive. Moreover, the ability of the surfaces to promote cell adhesion and proliferation often leads to higher risks of bacterial contamination.^44^ PEO layers are the focus of intensive research to generate surfaces resistant to bacteria using sources of inorganic additives such as Zn and Cu.^45,46^ Our data revealed that the Ti-A-350-100 sample showed minimum adhesion of bacteria both after short-time incubation with a suspension of bacteria and after longer 24 h incubation. On this surface, the lowest number of bacteria was recorded, lower even in comparison to the hydrophobic surface of the untreated (polished) Ti. Taking into account the higher corrosion resistance of this sample, it can be suggested that the corresponding PEO protocol can be used in the production of dental implants. Further optimization is required to determine the PEO parameters suitable for industrial production of Ti dental implants with increased bioactive and osseointegrative properties.

## 4 Conclusions

NTA can be used as a complexing agent in the PEO bath electrolyte to manufacture bioactive PEO surfaces on Ti implants. PEO-generated surfaces improve the adhesion and proliferation on titanium. Various PEO conditions resulted in separate improvements in the distinct biomedical aspects of the surfaces. The PEO parameters used to obtain the Ti-A-350-100 surface layer on the sample provide the most optimal conditions from the tested options in terms of the interaction between corrosion resistance, support of cell proliferation, and resistance to bacterial adhesion.

## 5 Experimental Procedures

### 5.1 Ti Specimens and PEO Processing

The samples were cut from a cylindrical rod of commercially available pure titanium (Grade 4; IWET, Kleosin, Poland) (diameter ϕ = 10 mm, thickness: 4 mm). Every sample was polished with abrasive SiC paper up to 1000 grit. Before PEO, each sample was degreased in an ultrasonic washer using isopropyl alcohol for 180 s, followed by rinsing in deionized water.

Titanium surfaces were oxidized using the PEO process (high-voltage dc power supply Kikusui PWR400H, Japan) at 300, 350, 400, and 450 V for 5 min. The PEO treatment was performed via dc galvanostatic anodization (anodic current density = 100 mA cm^–2^) up to limiting voltage. After the process voltage reached the limiting voltage, the treatment was conducted under a potentiostatic regime. The anodizing bath was placed in a glass electrolyzer, and during the PEO treatment, it was cooled with a glycol-filled cooling jacket, thermostatic at 10 °C. The treated titanium sample served as an anode, while a titanium mesh was used as a cathode. During the PEO process, the electrolyte was agitated with the use of a magnetic stirrer. After PEO, each sample was rinsed in deionized water and dried in air at room temperature. The electrolytes were composed of NTA, potassium hydrogen phosphate, and calcium formate (Table 7). Sample codes are presented in Table 8.

**Table 7:** Content of Bath Electrolytes Used in the Study

electrolyte	N(CH_2_CO_2_H)_3_, mol L^–1^	KH_2_PO_4_, mol L^–1^	Ca(HCOO)_2_, mol L^–1^
A	1.28	0.25	0.50
B	2.56	0.50	1.00

**Table 8:** Sample Codes (45 Samples for Each Group Were Prepared)

sample	electrolyte	voltage, V
Ti-A-350-100	A	350
Ti-A-450-150	A	450
Ti-B-300-100	B	300
Ti-B-400-100	B	400
Ti-B-450-100	B	450

### 5.2 Surface Characterization

#### 5.2.1 SEM with EDX Spectroscopy

Detailed examination of the chosen samples was carried out using the Phenom ProX microscope (Phenom-World BV, Eindhoven, The Netherlands) at an accelerating voltage of 15 kV. The chemical composition of the surface layer was analyzed by EDX spectroscopy using PhenomProX equipment.

#### 5.2.2 Roughness Assay

Roughness of the test material surfaces prepared by PEO was determined using a Surftest SJ-301 profilometer (Mitutoyo, Japan).

#### 5.2.3 Cross Section with EDX Mapping

The thickness and structure of the PEO coating layers were investigated using the cross-sectional method previously described^6^ with modifications. The PEO-coated Ti specimens were embedded in epoxy resin at room temperature. The embedded specimens were ground with rough silicon carbide (SiC) paper to remove the epoxy resin down to the Ti specimen. The grinding continued until the upper layer of PEO was removed to expose the bare Ti with the PEO-Ti border in the lateral plane and further polished using fine SiC paper up to 1500 grade. A thin layer of gold (Cressington Sputter Coater 108 Auto; Cressington Scientific Instruments UK, Watford, UK) was deposited on the sample, and the cross section was analyzed with SEM/EDX using Phenom Pro-X equipment at 15 kV. Image analysis was done with the Image-Pro 10.0.7 software.

#### 5.2.4 Raman Spectroscopy

A confocal Raman microscope (inVia Renishaw, Gloucestershire, UK) equipped with a CCD detector was used with a green laser (514 nm) for excitation. An extended range of scans (100–350,000 cm^–1^) was taken. Raman spectra were measured at 10 randomly selected locations for each sample (*n* = 10). The spectra presented in Figure 2 are the arithmetic mean of the measurements made. The relative error of the measurements did not exceed 0.25%. The measurements were made in a backscattering geometry, using a 50× microscope objective with an aperture of 0.75, giving scattering areas of about 1 μm^2^. Single-point spectra were recorded with 4 cm^–1^ resolution, 30 s accumulation with 8 data acquisitions.

#### 5.2.5 X-ray Photoelectron Spectroscopy

XPS measurements were made using a PHI 5000 VersaProbe (ULVAC-PHI Inc., Hagisono, Chigasaki, Kanagawa, Japan) spectrometer with monochromatic Al Kα radiation (*h*ν = 1486.6 eV) from an X-ray source operating at 100 μm spot size, 25 W, and 15 kV. High-resolution (HR) XPS spectra were collected with a hemispherical analyzer at a pass energy of 117.4 and an energy step size of 0.1 eV. The X-ray beam was incident on the sample surface at the angle of 45° with respect to the surface normal, and the analyzer axis was located at 45° with respect to the surface. The Thermo Avantage software (version 5.988) was used to evaluate the XPS data. Deconvolution of all HR XPS spectra was performed using a Shirley background and a Gaussian peak shape with 30% Lorentzian character. The identification of the chemical state of the elements detected in the sample was carried out based on the literature data and electronic XPS databases. The measurements were corrected against a carbon peak of 284.5 eV.

#### 5.2.6 X-ray Diffraction

X-ray diffraction (XRD) experiments were carried out as described in ref (17) using the X’Pert PW 3040/60 equipment (Philips, Amsterdam, the Netherlands) at 30 mA and 40 kV. XRD patterns were recorded in the 2θ region: 10–140°using CuKα_1,2_ radiation (λCu Kα_1_ = 1.54056 and λCu Kα_2_ = 1.54443 Å) for the incident α angle of 0.25°.

### 5.3 Corrosion Resistance Investigations

#### 5.3.1 Electrochemical Corrosion Assay

Resistance to electrochemical corrosion was measured in a 250 mL glass bottle filled with Ringer solution (8.6 g L^–1^ NaCl, 0.3 g L^–1^ KCl, and 0.48 g L^–1^ CaCl_2_·6H_2_O; Fresenius Kabi, Warsaw, Poland) at 37 °C. The Ringer solution, before testing, was kept in an incubator at 37.0 ± 0.5 °C to avoid the formation of gas bubbles in the corrosion cell during heating to the measurement temperature. The experiments were performed in the three-electrode configuration. The test sample (prepared from a rod with a diameter of 8 mm) was a working electrode (a gasket with a diameter of 6 mm; test surface = 0.283 cm^2^), a SCE with a Haber-Luggin capillary was used as a reference electrode, while a platinum mesh was used as the counter electrode. The measurement procedure was prepared using the VersaStudio version 2.60.6 software, which was used to operate the PARSTAT 4000A potentiostat–galvanostat (Princeton Applied Research, Ametek, Berwyn, PA, USA). The total measurement time for one sample was approximately 3 h 45 min. For all surfaces, samples were tested in duplicate to ensure the reproducibility of the measurements.

#### 5.3.2 Long-Term Ringer Corrosion Assay

The tests were carried out in an incubator at 37 °C in 20 mL of Ringer solution on a shaker at 60 rpm for 12 weeks. The release of Ti, Ca, and P was determined with inductively coupled plasma-optical emission spectroscopy (ICP-OES) with a Varian 710-ES spectrometer (Varian Inc., Palo Alto, CA, USA) using parameters as described.^17^

### 5.4 In Vitro Characterization

#### 5.4.1 Cytotoxicity and Cell Viability Assays

The samples were autoclaved at 121 °C for 1 h and placed on a 24-well sterile plate. 2 mL of 20% fetal bovine serum (FBS, Invitrogen) in Dulbecco modified Eagle medium (DMEM; Invitrogen, cat. no. 11960) was added to each sample to mimic protein adsorption on the surfaces of the implants within the body environment after implantation. After 24 h of incubation, DMEM/FBS was removed and U2OS rat osteoblast (obtained from Sumy State University) in amounts of 10E5 cells were seeded on the upper surface of each sample. 2 mL of complete medium containing DMEM supplemented with 10% FBS, 2 mM l-glutamine (Invitrogen, cat. no. 25030), 0.1 mM 2-mercaptoeth,anol (Sigma, cat. no. M7522), 50 units mL^–1^ penicillin, and 50 units mL^–1^ streptomycin (Invitrogen, cat. no. 15070) was added to each well and incubated at 37 °C in a humidified environment with 5% CO_2_. The culture medium was changed every 3 days for a 7 day period. All experiments were carried out in triplicate. Wells with cells only and polished Ti samples (reference sample) were used as controls. Cell viability and proliferation were measured with a resazurin reduction assay as described.^14^ Data were calculated using the formula of the Method for Measuring Cytotoxicity or Proliferation Using AlamarBlue by Spectrophotometry (BioRad)^47^ on days 1, 3, and 7.

For the assessment of cell distribution on the surface, DAPI (Roche) was used. After the 7th day of osteoblast cultivation, the samples were washed with PBS and incubated with DAPI in PBS for 2 min, followed by washing with PBS. The scaffolds were placed on glass slides and analyzed with a fluorescence microscope (Axio Imager A1 microscope, Carl Zeiss) in the DAPI channel. Fluorescent staining and analysis were performed in the SUMEYA Ukrainian-Swedish Research Center (Sumy, Ukraine).

#### 5.4.2 Bacterial Adhesion Assay

The adhesive properties of the processed Ti samples were evaluated with the Gram-positive bacterium *Staphylococcus aureus* strain B 918. The bacteria grown on nutrient agar at 37° C for 24 h was collected with a loop, suspended in saline (0.9%, w/v NaCl), and the concentration was adjusted to 10E5 colony-forming units (CFUs) mL^–1^ (5 log CFU) in nutrient broth using McFarland standards. Samples were horizontally incubated with 2.0 ml of the bacterial suspension under static conditions in a 24-well plate at 37 °C for 2, 4, 6, and 24 h. The samples were removed with sterile forceps and rinsed with 2.0 mL of sterile saline three times to remove loosely adherent bacteria. Subsequently, the disks were placed in sterile tubes with 1.0 mL of sterile saline and sonicated for 1 min in an ultrasonic bath (B3500S-MT, Bransone Ultrasonics Co., Shanghai, China) to remove adherent bacteria from the surfaces. 10 μL aliquots were placed on nutrient agar using the streak plate technique, and the colonies were counted after 24 h of incubation at 37 °C^.^ All experiments were carried out in triplicate.

## Supporting Information Available

Additional experimental details and results, including details of roughness profiling of the sample Ti-B-450-100; EDX spectra of the selected elements in the PEO samples; chemical states of elements in the oxide coating on the sample Ti-A-350-100; chemical states of elements in the oxide coating on the sample Ti-B-400-100; PDP curves; electrochemical impedance spectra; and equivalent electrical circuits not included in the main text (PDF)

## Notes

^○^ Shared correspondence authorship.
