# Supplementary material for: Nitrilotriacetic Acid Improves Plasma Electrolytic Oxidation of Titanium for Biomedical Applications
Source: ACS Appl Mater Interfaces. 2023 Apr 11;15(16):19863–76. doi: 10.1021/acsami.3c00170 (PMC10141263; doi:10.1021/acsami.3c00170)
Supplement: Supplementary file 1 — am3c00170_si_001.pdf [file am3c00170_si_001.pdf]

Supporting information

Nitrilotriacetic acid improves plasma electrolytic oxidation of titanium for biomedical applications

Sergiy Kyrylenko[1], Maciej Sowa[2], Alicja Kazek-Kęsik[2], Agnieszka Stolarczyk[2], Marcin Pisarek[3], Yevheniia Husak[1,2], Viktoriia Korniienko[1,4], Volodymyr Deineka[1], Roman Moskalenko[5], Izabela Matuła[6], Joanna Michalska[2], Agata Jakóbik-Kolon[2], Oleg Mishchenko[7,8], Maksym Pogorielov[1,4,£], Wojciech Simka[2,*,£]

[1] Sumy State University, Biomedical Research Center, 31 Sanatorna St, Sumy 40018, Ukraine

[2] Silesian University of Technology, Faculty of Chemistry, 6 B. Krzywoustego St, 44-100 Gliwice, Poland

[3] Institute of Physical Chemistry PAS, M. Kasprzaka str. 44/52, 01-224 Warsaw, Poland

[4] University of Latvia, Institute of Atomic Physics and Spectroscopy, 3 Jelgavas St, Riga LV-1004, Latvia

[5] Sumy State University, Ukrainian-Swedish Research Center SUMEYA, 31 Pryvokzalna St, Sumy 40018, Ukraine

[6] University of Silesia, Faculty of Science and Technology, Institute of Materials Engineering, 75 Pułku Piechoty St. 1a, 41-500 Chorzów, Poland

[7] Nano Prime LTD, 25 Metalowców St, 39-200 Dębica, Poland

[8] Zaporizhzhia State Medical University, 26 Maiakovskyi Ave, 69035, Zaporizhzhia, Ukraine

*Address correspondence to: wojciech.simka@polsl.pl (W.S.), m.pogorielov@gmail.com (M.P.)

[£] Shared correspondence authorship

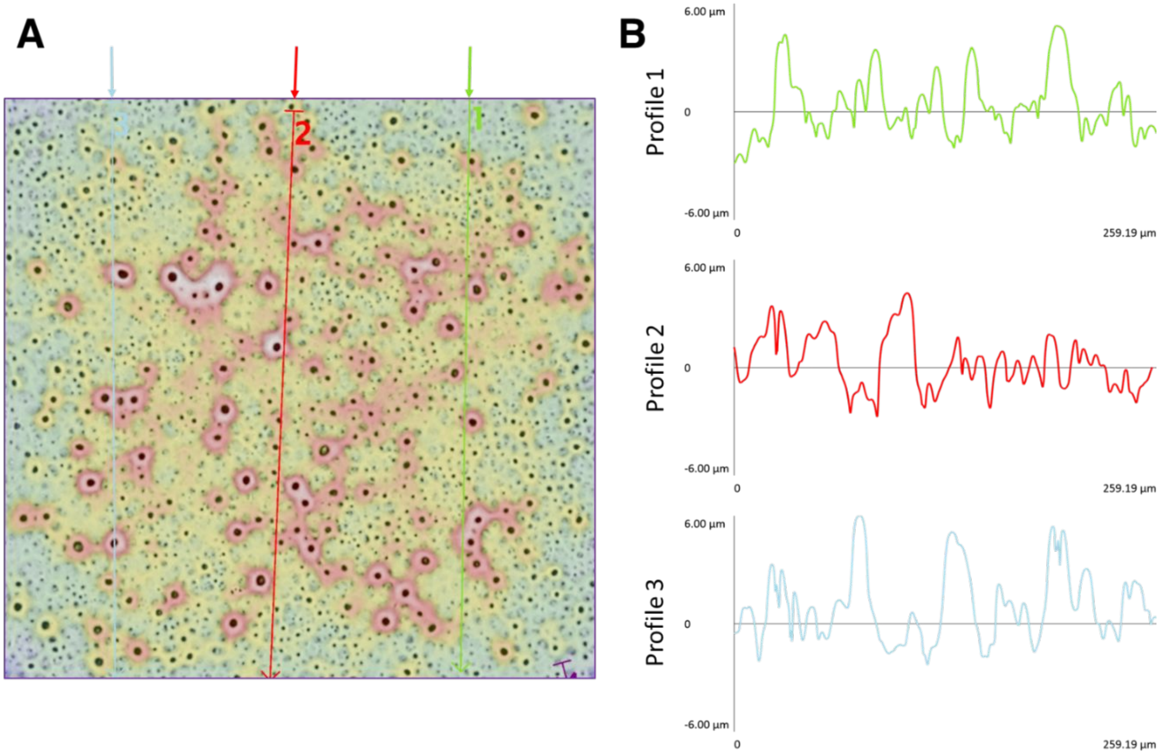

Supplementary Figure S1. Details of roughness profiling of the selected sample Ti-B-450-100. A, field view with 3 profiling lines indicated; B, corresponding profiles.

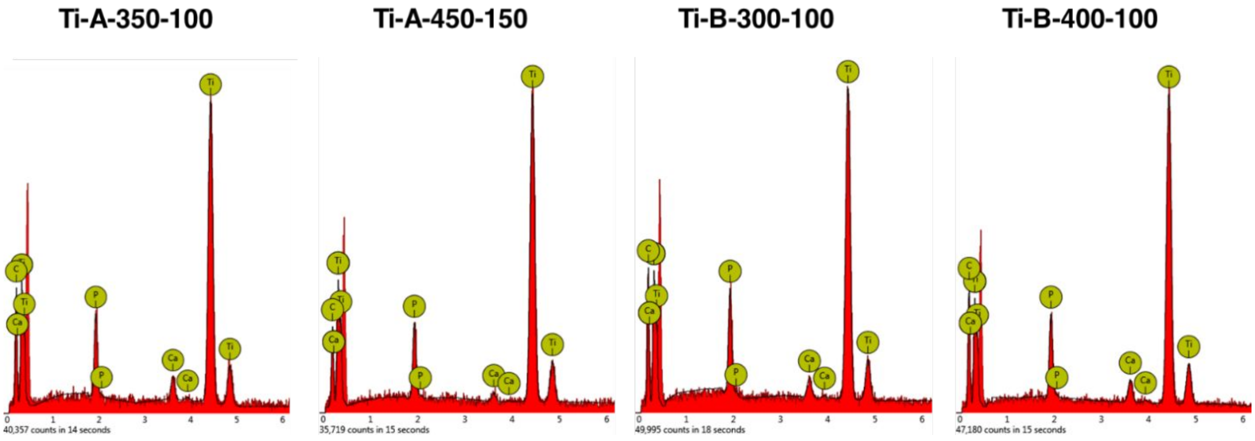

Supplementary Figure S2. The EDX spectra of the selected elements in the PEO samples as indicated.

Supplementary Table S1. Chemical states of elements in the oxide coating on sample Ti-A-350-100.

	BE / eV	At. %	SF	BE / eV (+0.71 eV)	Chemical state
P2p3	132.4	10.0	0.896	**133.1**	phosphates
P2p1	133.3	0.0	0.457	134.0	
C1s	283.8	15.2	1	**284.5**	C-C
C1s	285.5	2.4	1	**286.2**	C-O / C-N
C1s	287.9	1.5	1	**288.6**	O=C-OH
Ca2p3	346.6	0.5	3.944	**347.3**	calcium phosphate
Ca2p1	350.2	0.0	2.027	350.9	
N1s	399.1	1.0	1.676	**399.8**	$C\text{-}NH_2$
N1s	400.9	0.6	1.676	**401.6**	$C\text{-}NH_3^*$
Ti2p3	458.0	14.6	4.415	**458.7**	Ti-O (TiO_2)
Ti2p1	463.7	0.0	2.055	464.4	
O1s	530.4	27.7	2.881	**531.1**	phosphates
O1s	529.3	17.8	2.881	**530.0**	metal oxides
O1s	531.2	6.0	2.881	**532.0**	C=O
O1s	532.3	2.6	2.881	**533.0**	C-O
K2p3	291.7	0.1	3.081	**292.5**	potasium phosphate
K2p1	294.3	0.0	1.59	295.0	
		**100,0**			

Supplementary Table S2. Chemical states of elements in the oxide coating on sample Ti-B-400-100.

	BE / eV	At. %	SF	BE / eV (+0.51 eV)	Chemical state
P2p3	132.5	9.4	0.896	**133.0**	phosphates
P2p1	133.2	0.0	0.457	133.7	
P2p3	133.7	1.4	0.896	**134.3**	metaphosphates
P2p1	134.3	0.0	0.457	134.8	
C1s	284.0	23.9	1	**284.5**	C-C
C1s	285.7	1.9	1	**286.2**	C-O / C-N
C1s	288.1	1.3	1	**288.6**	O=C-OH
K2p3	292.0	0.3	3.081	**292.5**	potasium phosphate
K2p1	294.8	0.0	1.59	295.3	
Ca2p3	346.7	2.8	3.944	**347.2**	calcium phosphate
Ca2p1	350.3	0.0	2.027	350.8	
N1s	399.1	0.7	1.676	**399.6**	$C-NH_2$
N1s	401.2	0.4	1.676	**401.7**	$C-NH_3^*$
Ti2p3	458.2	10.4	4.415	**458.7**	Ti-O (TiO_2)
Ti2p1	463.9	0.0	2.055	464.4	
O1s	530.5	29.1	2.881	**531.0**	phosphate
O1s	529.5	12.1	2.881	**530.0**	metal oxides
O1s	531.6	4.3	2.881	**532.1**	C=O
O1s	532.6	2.1	2.881	**533.1**	C-O
		**100,0**			

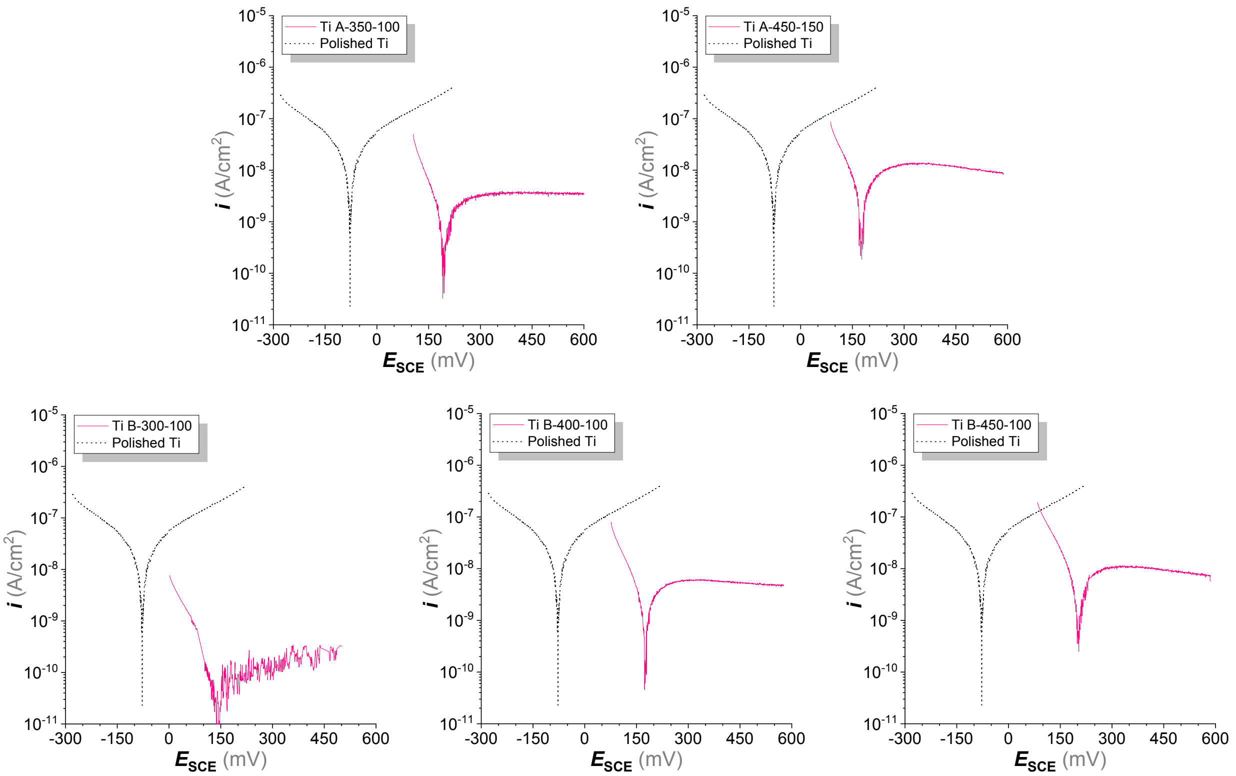

Supplementary Figure S3. Potentiodynamic polarization curves measured for the titanium specimens subjected to electrochemical treatment. In each plot, the curve corresponding to the Polished Ti sample has been added for reference.

i, absolute value of current density flowing through the sample; E_{SCE}, applied potential with respect to saturated calomel electrode

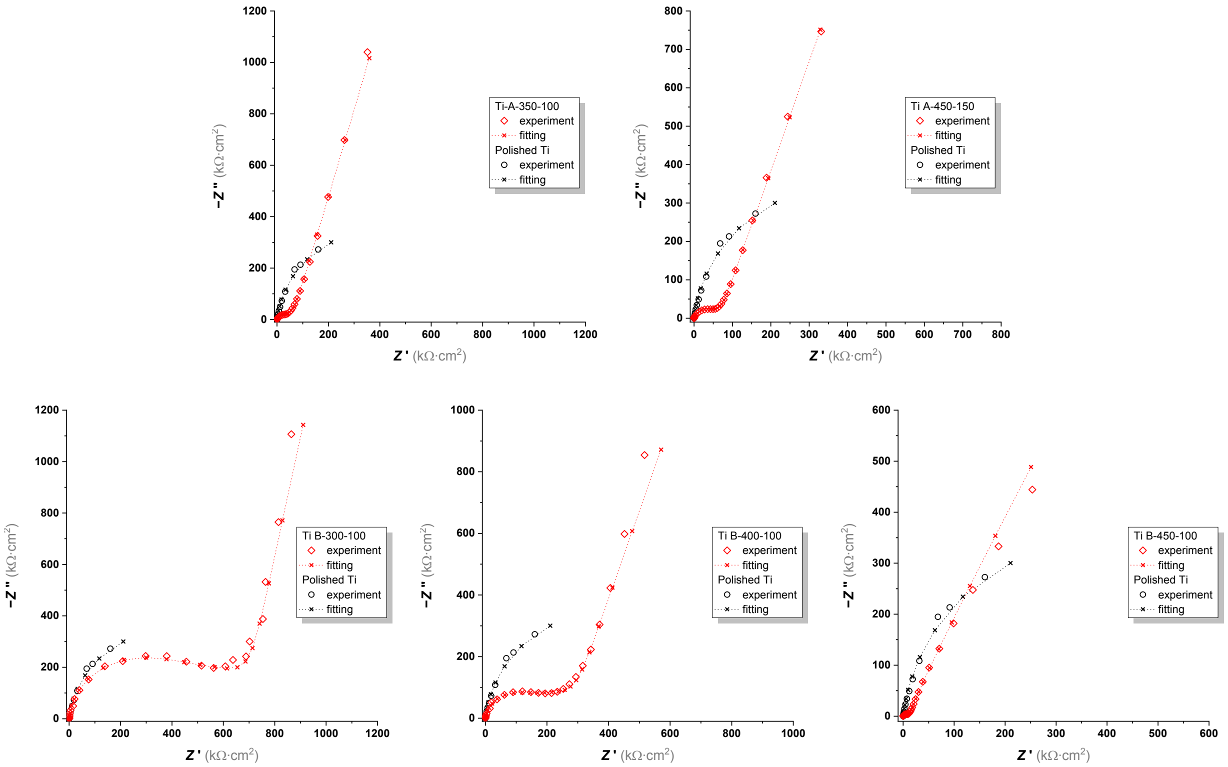

Supplementary Figure S4. Nyquist plots showing electrochemical impedance spectra measured for the titanium specimens subjected to electrochemical treatment. In each plot, the spectrum corresponding to the Polished Ti sample has been added for reference. The measured impedance was normalized with respect to the surface area of the sample exposed to the corrosion medium. *Z*', real part of complex impedance; *Z*'', imaginary part of complex impedance.

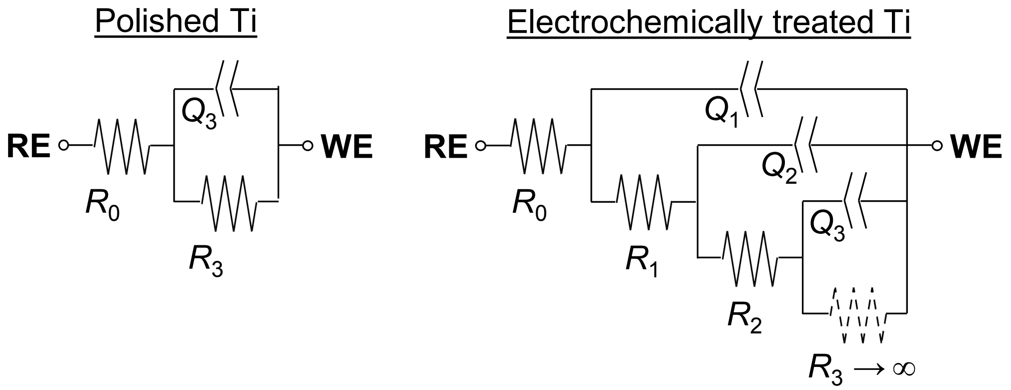

Supplementary Figure S5. Equivalent electrical circuits used to fit the electrochemical impedance results. R_3 in the case of the electrochemically treated Ti was assumed to approach infinity in order to properly determine the other parameters of the chosen circuit. RE, reference electrode; WE, working electrode
